# The effect of a four-week intensive scoliosis-specific exercises programme on Cobb angle in subjects with idiopathic scoliosis: an 11 patient case series

**DOI:** 10.1186/1748-7161-7-S1-O51

**Published:** 2012-01-27

**Authors:** E Maude, J Head, K Hobson

**Affiliations:** 1 Scoliosis SOS Clinic, Martlesham, UK

## Background

Current management for scoliosis in the UK is dictated by a patient’s Cobb angle. This case series aims to investigate whether a four-week intensive scoliosis-specific exercise programme results in a significant improvement in patient’s Cobb angle measurements.

## Materials and methods

11 patients (9 females, 2 male) with IS (9 thoracic curve patients and 5 thoracolumbar/lumbar curve patients) and a mean age of 16.45 years (Range 7-36 years) were treated with a four-week intensive scoliosis-specific physiotherapy course (ScolioGold). Patients’ initial x-rays were supplied in retrospect and follow-up x-rays gathered after patients’ routine check-up appointments. Each x-ray was taken by an independent radiographer and all x-rays measured by the same rater.

## Results

Mean thoracic Cobb angle before treatment was 44.89° (SD12.41°, Range 19°-60°) while mean thoracolumbar/lumbar Cobb angle before treatment was 45.60° (SD5.55°, Range 41°-55°), post treatment this reduced to 36.22° (SD12.17°, Range 10°-50°) for thoracic curves and 33.80° (SD9.42°, Range 21°-44°) for thoracolumbar/lumbar curves. This is a reduction in thoracic curves of 8.89° (SD2.37° Range 5°-12°) conversely this equates to a 21.96% reduction (SD11.23%, Range 12.2%-47.37%) and 11.8° in thoracolumbar/lumbar curves (SD5.85° range 4°-20°) conversely this equates to a 26.64% reduction (SD 15.11%, range 8.89%-48.78%).

## Conclusions

With the significance level for Cobb angle reduction set at 5° degrees, these changes show a significant improvement in this case series. Results substantiate the use of intensive exercise methods (ScolioGold) in the treatment of IS patients with the aim to reduce the Cobb angle and add to the growing body of evidence for scoliosis-specific physiotherapy.

